# Transgenerational Transcriptomic and DNA Methylome Profiling of Mouse Fetal Testicular Germline and Somatic Cells after Exposure of Pregnant Mothers to Tributyltin, a Potent Obesogen

**DOI:** 10.3390/metabo12020095

**Published:** 2022-01-20

**Authors:** Keiko Shioda, Junko Odajima, Bruce Blumberg, Toshi Shioda

**Affiliations:** 1 Center for Cancer Research, Massachusetts General Hospital, Building 149, 13th Street, Charlestown, MA 02129, USA; kshioda@mgh.harvard.edu (K.S.); jodajima@mgh.harvard.edu (J.O.); 2 Department of Developmental and Cell Biology, University of California, Irvine, CA 92697-2300, USA; blumberg@uci.edu; 3 Department of Pharmaceutical Sciences, University of California, Irvine, CA 92697-2300, USA; 4 Center for Cancer Research, Massachusetts General Hospital, Harvard Medical School, Building 149, 13th Street, Charlestown, MA 02129, USA

**Keywords:** transgenerational, obesity, obesogen, diet-induced obesity, tributyltin, RNA-seq, MBD-seq, primordial germ cells

## Abstract

Obesogens such as tributyltin (TBT) are xenobiotic compounds that promote obesity, in part by distorting the normal balance of lipid metabolism. The obesogenic effects of TBT can be observed in directly exposed (F1 and F2 generations) and also subsequent generations (F3 and beyond) that were never exposed. To address the effects of TBT exposure on germ cells, we exposed pregnant transgenic OG2 mouse dams (F0), which specifically express EGFP in germline cells, to an environmentally relevant dose of TBT or DMSO throughout gestation through drinking water. When fed with a high-fat diet, F3 male offspring of TBT-exposed F0 dams (TBT-F3) accumulated much more body fat than did DMSO-F3 males. TBT-F3 males also lost more body fluid and lean compositions than did DMSO-F3 males. Expression of genes involved in transcriptional regulation or mesenchymal differentiation was up-regulated in somatic cells of TBT-F1 (but not TBT-F3) E18.5 fetal testes, and promoter-associated CpG islands were hyper-methylated in TBT-F1 somatic cells. Global mRNA expression of protein-coding genes in F1 or F3 fetal testicular cells was unaffected by F0 exposure to TBT; however, expression of a subset of endogenous retroviruses was significantly affected in F1 and F3. We infer that TBT may directly target testicular somatic cells in F1 testes to irreversibly affect epigenetic suppression of endogenous retroviruses in both germline and somatic cells.

## 1. Introduction

Perinatal exposure to a subset of endocrine-disrupting chemicals (EDCs) distorts the normal balance of lipid metabolism to promote obesity later in life [1]. Such EDCs are collectively referred to as *obesogens* [1,2,3]. Studies using laboratory rodent models revealed that the metabolic disorders caused by obesogens can be transmitted to generations that have never been directly exposed to the chemicals [3]. When a pregnant rodent dam is exposed to a xenobiotic chemical, the F1 fetuses are exposed in utero. Importantly, germline cells already developing in the gonads of F1 fetuses are also directly exposed to the chemical. Thus, health impact phenotypes observed with the F1 and F2 generations may be attributed to the direct exposure. However, when animals of the F3 generations or beyond show phenotypes, the effects of chemical exposure are considered inherited. The phenotypes observed with offspring that were exposed to toxicants are commonly mentioned as *multigenerational* whereas effects observed with generations never exposed to chemicals are *transgenerational* [4].

The mechanisms underlying transgenerational inheritance of EDC effects in mammals is still a debatable subject [3]. Our preceding studies established a mouse model suitable for mechanistic investigations of the transgenerational obesogenic effects of tributyltin (TBT). TBT is a representative obesogen and a potent EDC that activates the nuclear hormone receptors RXR and PPARγ that act as a heterodimer to regulate adipocyte differentiation [2,3,5,6,7,8]. When pregnant C57BL/6 female mice (F0 generation) were exposed to an environmentally relevant, low dose of TBT via drinking water throughout pregnancy [6,9] or throughout pregnancy and lactation [5], F3 and F4 male offspring were more susceptible to diet-induced obesity than control animals whose F0 ancestor dams were gestationally exposed to DMSO vehicle [5,6,9]. The transgenerationally transmitted propensity for diet-induced obesity was observed only with F3/F4 male offspring with no significant effects on females of the same generations [5,6,9]. DNA methylome profiling provided evidence of altered chromatin organization in sperm and white adipose tissues that appeared to be caused by the ancestral exposure to TBT [5,7]. Integrated analyses of gDNA methylation and transcriptomes supported the hypothesis that environmental stressors like TBT can cause a self-propagating disruption of chromatin organization and that such disruptions are reconstructed in subsequent generations [7].

To obtain further insight into the epigenetic basis of the transgenerationally transmittable predisposition to diet-induced obesity after gestational exposure to TBT, our current study employed the OG2 transgenic mouse line in which prenatal germline cells specifically express EGFP, allowing efficient separation of germline cells and somatic cells in total testicular cell population. We first demonstrated the reproducibility of the transgenerational obesity created after gestational exposure of F0 dams to TBT and then present evidence that the testicular somatic cells of the F1 fetuses are significantly affected by the F0 exposure to TBT but not those of the F3 fetuses. Expression of mRNAs from protein-coding genes in fetal testicular germline cells was not significantly affected in the F1 or F3 generation. However, expression of a subset of mouse endogenous retroviruses (mERVs) that are normally suppressed in fetal testicular germline cells by epigenetic mechanisms involving the MORC1 chromatin suppression gene showed persistent expression after the gestational exposure of F0 dams to TBT. These results suggested that exposure to TBT disrupts the integrity of heterochromatin structures that normally suppress expression of transposons in the healthy germline genome. Moreover, such aberrations may not be immediately repaired in the subsequent generations, potentially contributing to the transgenerationally transmittable predisposition to diet-induced obesity.

## 2. Results

**Transgenerational diet-induced obesity initiated by in utero exposure of mice to TBT.** We previously reported that in utero exposure of C57BL/6J mice to an environmentally relevant dose of TBT initiated transgenerationally inherited predisposition to diet-induced central obesity [5,6,7,9]. To obtain mechanistic insights into this phenomenon, in the current study, we repeated the animal exposure experiment using the OG2 transgenic mice, which express EGFP specifically in primordial germ cells and prenatal germline precursor cells driven by the *Pou5f1*/Oct4 promoter and distal enhancer [10].

**Figure 1:**
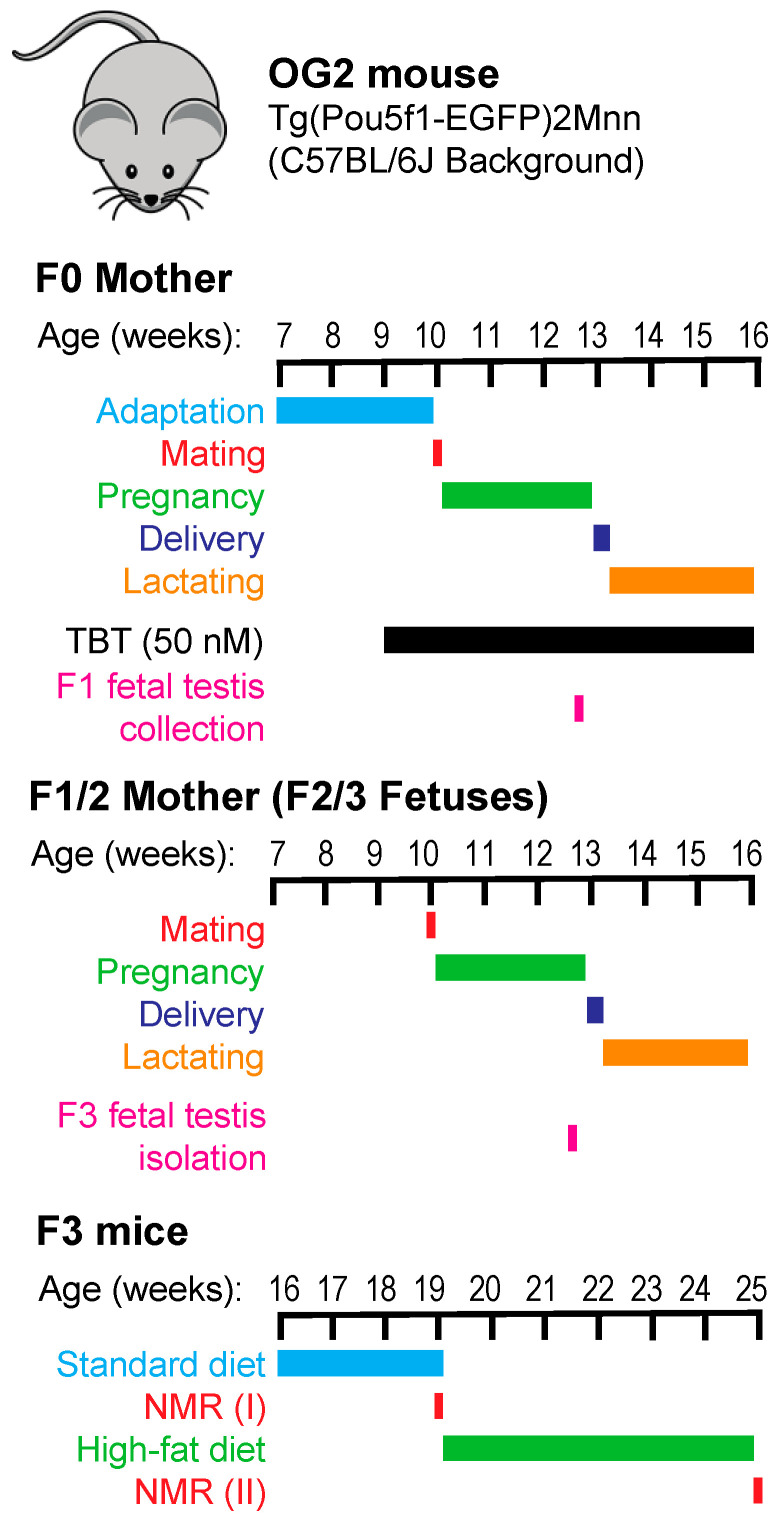
Timelines of breeding, exposure, diet challenge, NMR assessments of body compositions, and collection of fetal gonads.

Breeding, exposure, and sample collection scheme are shown in Figure 1. Female OG2 mice (F0 mothers) were exposed to TBT through drinking water from one week prior to mating and throughout pregnancy and lactation until pups were weaned three weeks after birth. Following our previously published mating scheme [5,6], F1 pups of the TBT-exposure F0 mothers were mated to obtain TBT-F2 animals. In this breeding, siblings were not mated, and a male was mated with not more than one female belonging to each litter. F1 pups of the control, DMSO-exposed F0 mothers were mated separately to obtain DMSO-F2 animals. F3 animals were obtained by similarly breeding F2 mice. Fetal testes were collected from F1 and F3 males at E18.5 dpc to separate the EGFP-positive germline cells and EGFP-negative somatic cells by FACS. F3 animals were fed with a standard chow (STD; 14% calories derived from fat) until 19-weeks-old and then challenged with a high-fat diet (HF; 21.6% calories derived from fat) for 6 weeks. Energies of the standard and HF chows were comparable—namely, 3.20 and 3.45 kcal/g respectively. Total body weight and body compositions of F3 animals were determined before and after the HF diet challenge.

**Figure 2:**
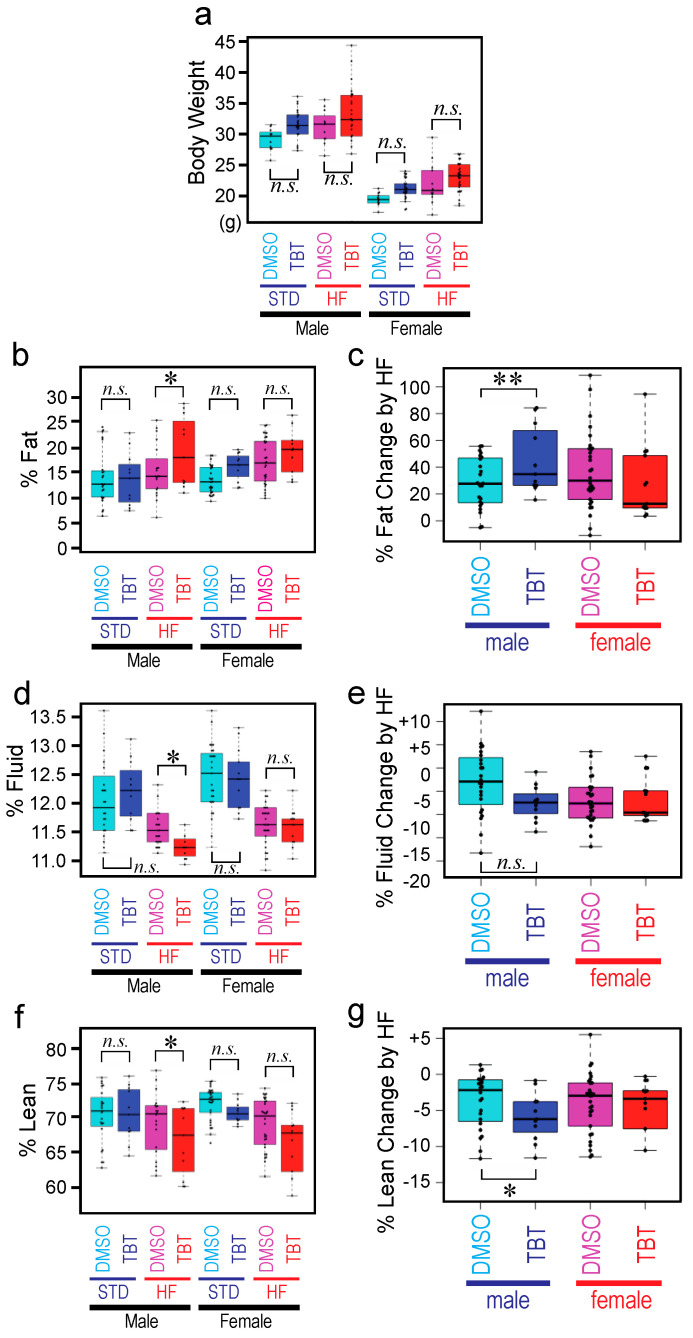
Diet-induced obesity of TBT-exposed F3 mice. F3 offspring of F0 mothers gestationally exposed to TBT or DMSO were fed with a standard (STD) diet until 19-weeks-old and then challenged with a high-fat (HF) diet for 6 weeks. Total body weight or body compositions were measured before and after the HF diet. Each box plot represents the median, 25/75 percentile, and min/max of data (*n* = 11~30). Statistical significance (*t*-test with Welch’s correction for heteroscedastic data pairs) is indicated by *, *p* < 0.05; **, *p* < 0.01, *n.s.,* not significant. (**a**) Total body weight. (**b**–**g**) NMR assessments of fat (**b**,**c**), fluid (**d**,**e**), and lean (**f**,**g**). Panels (**c**,**e**,**f**) show relative changes by HF challenges in fat, fluid, and lean, respectively.

Exposure of F0 mothers to TBT did not affect fecundity or sex ratio of animals in the F1, F2, or F3 generations. We did not observe apparent deterioration of health for any animals throughout the experiment. Total body weight of the F3 animals (both males and females) whose F0 mother was exposed to TBT (TBT-F3) or DMSO (DMSO-F3) did not show significant difference at the end of the STD diet feeding or after the subsequent 6week HF diet challenge (Figure 2a), reproducing our previous observations [5,6]. Body composition analysis was performed using the non-invasive, Time Domain (TD) NMR technology [11] to determine percent body fat (Figure 2b), fluid (Figure 2d), and lean (Figure 2f). We observed significant increase in fat of the TBT-F3 males compared to the DMSO-F3 males only after the HF diet challenge (Figure 2b). Fat of TBT-F3 males and DMSO-F3 males did not differ when fed with the STD diet (Figure 2b). TBT-F3 females and DMSO-F3 females had equal amounts of fat, and the HF diet challenge did not cause a significant difference (Figure 2b). On the other hand, compositions of body fluid and lean in TBT-F3 males was significantly smaller than those in DMSO-F3 males only after the HF diet challenge (Figure 2d,f). Again, fluid or lean of TBT-F3 males did not differ from those of DMSO-F3 males when fed with the STD diet (Figure 2d,f). Fluid or lean of TBT-F3 females and DMSO females are equal with no effect by the HF diet challenge.

Taking advantage of the non-invasive NMR determination of body compositions, we were able to compare body fat, fluid, and lean of the same animal immediately before and after the HF diet challenge. Figure 2c,e,g shows relative changes in body compositions for fat, fluid, and lean, respectively. For each animal, the value before the HF diet challenge was subtracted from the value after the challenge, and the difference was divided by the value before the challenge. Thus, when the body fat composition is 13% and 16% before and after the HF diet challenges, the *% Fat Change by HF* value is (16–13)/13 = 0.23 (i.e., 23%). The HF diet-induced increase in body fat composition of TBT-F3 males was greater than that of DMSO-F3 males (Figure 2c). In contrast, the HF diet-induced decrease in body lean composition of TBT-F3 males was greater than that of DMSO-F3 males (Figure 2g). Although not reaching the statistical significance (*p*-value cutoff = 0.05), body fluid composition showed a similar decrease as body lean (Figure 2e). Body compositions of TBT-F3 females and DMSO-F3 females did not show differences in their responses to the HF diet challenge (Figure 2c,e,g).

Thus, our present experiment performed using the OG2 mice largely reproduced our preceding studies [5,6], demonstrating that in utero exposure of mice to an environmentally relevant, low dose of TBT causes predisposition to HFD-inducible normal-weight obesity. The normal-weight obesity syndrome is a non-classical type of metabolic disorder of humans characterized with excess amounts of body fat while the body mass index is maintained within the normal range. This syndrome is linked to increased cardiovascular mortality and morbidity as a significant human health threat [12].

**Figure 3:**
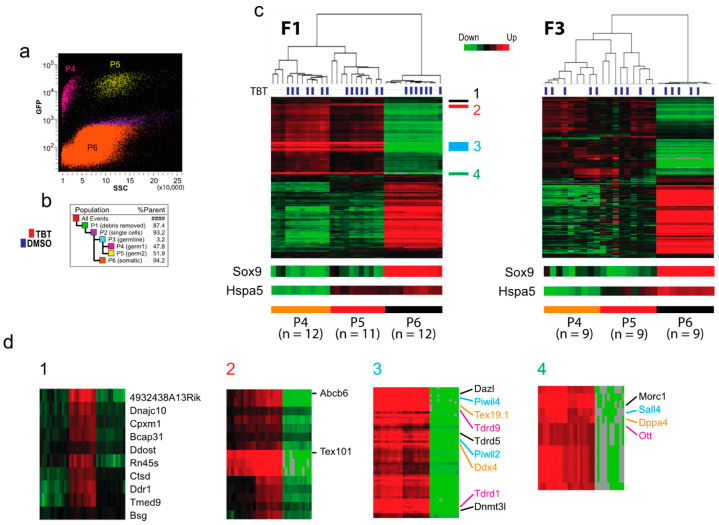
Transcriptomes of F1 and F3 fetal testicular cells. (**a**,**b**) FACS enrichment of EGFP^+^ germline cells (P4 and P5) and EGFP^-^ somatic cells (P6) from E18.5 fetuses. (**a**) Germline and somatic cell populations isolated by FACS with GFP and side scatter (SSC) intensities. (**b**) Relative sizes of cell populations. (**c**,**d**) Heatmap representations of unsupervised hierarchical clustering of transcriptomes determined by RNA-seq for F1 and F3 testicular cells. Transcriptomes of animals whose F0 mother was exposed to TBT are labelled by blue rectangles. The green-red colors indicate down- and up-regulation of genes. Parts of transcriptomes in panel (**c**) are enlarged in panel (**d**).

**Transcriptomic impact of F0 exposure to TBT on testicular cells of the offspring.** To obtain insights into possible transcriptomic alterations associated with the transgenerational transmission of the TBT-induced obesity predisposition, we isolated testicular germline and somatic cells from E18.5 F1 and F3 fetuses by FACS and evaluated the mRNA transcriptomes with RNA-seq. Total testicular cell population was divided into EGFP-positive germline cell populations (P4 and P5, together ~3%) and an EGFP-negative somatic cell population (P6, 94%) (Figure 3a,b). The EGFP-positive germline cells were separated further to nearly equal sizes of populations P4 and P5 by their difference in side scatter.

Unsupervised hierarchical clustering of RNA-seq data confirmed that both P4 and P5 populations represented germline cells as they commonly and strongly expressed germline marker genes Dazl, Ddx4, Dppa4, and Sall4 (Figure 3c,d2–4) [13]. The PIWI-like proteins PIWIL2, PIWIL4, and the tudor domain-containing proteins Tdrd1, Tdrd5, and Tdrd9, and MORC1 play a critical role in suppression of transposon expression in germline cells [14,15]. Testis-specific genes Tex101 and Tex19.1 are expressed specifically in the germline cells during spermatogenesis [16,17]. A small number of genes, including Hspa5, were differentially expressed between P4 and P5 populations (Figure 3c,d1). As Hspa5 is involved in sperm maturation in mice [18], we presume that P4 and P5 represent testicular germline cells in different stages of spermatogenesis, P4 being more advanced than P5. Because expression of the germline marker genes in P6 is very weak (Figure 3c,d), we presume that contamination of germline cells in the P6 population of somatic cells is too low to impact the transcriptomal analysis. Whereas this unsupervised analysis clearly separated P4, P5, and P6 populations, it did not separate testes derived from TBT- or DMSO-groups (Figure 3c), indicating that TBT exposure of F0 mothers did not significantly impact the global expression of protein-coding genes in the F1 and F3 germline cells or testicular somatic cells. Principal Component Analysis (PCA) of RNA-seq data (Figure S1a,b) also showed clear separation between the germline cells (P4 and P5 combined) and somatic cells (P6) whereas TBT- and DMSO-groups are intermingled.

**Figure 4:**
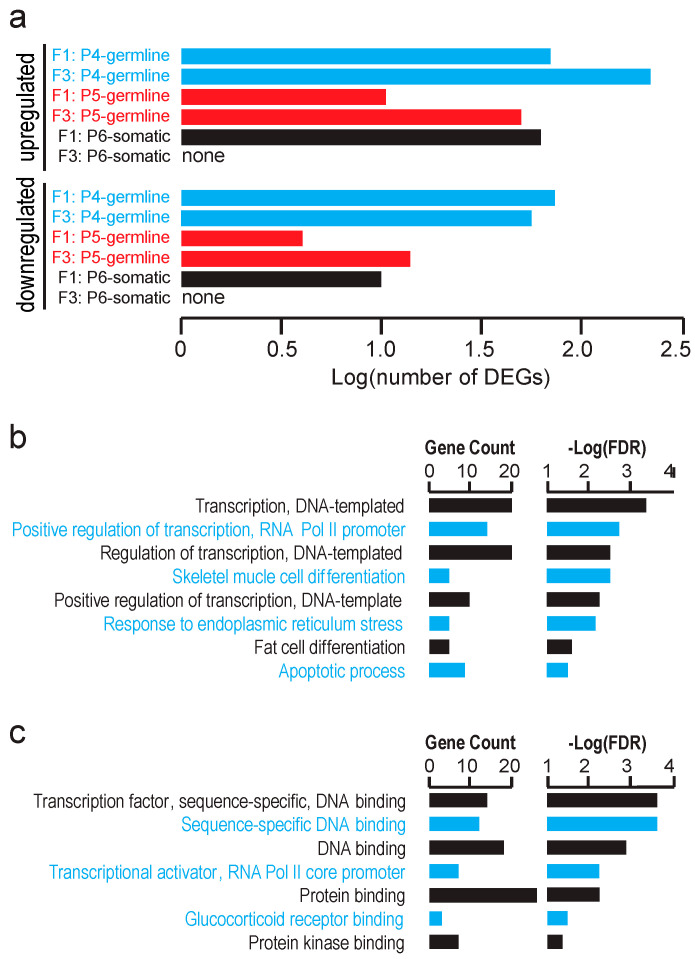
Differentially expressed genes (DEGs) in fetal testes. (**a**) Numbers of genes up- or down-regulated in E18.5 F3 fetal testicular cells of the TBT-exposed lineage compared to the DMSO-exposed control lineage. (**b**,**c**) Gene ontology analysis of DEGs up-regulated by TBT exposure in F1 P6-somatic cells; (**b**) biological pathways, and (**c**) molecular functions.

Since we did not detect significant global transcriptomic effects of the gestational F0 mother exposure to TBT on testicular cells of the F1 or F3 male offspring, we attempted to identify individual genes differentially expressed between the TBT- and DMSO-offspring testes. Using false discovery rate (FDR) < 0.1 as a relatively inclusive cutoff criterion, we were able to identify up-regulated and down-regulated genes in the FACS-separated populations of testicular cells in E18.5 F1 and F3 fetuses although no differentially expressed gene (DEG) was identified for the P6 somatic cell population in the F3 male fetuses (Table S1). Numbers of these DEGs are shown in Figure 4a. Gene ontology (GO) analysis did not detect any statistically significant enrichment of biological pathways for these DEGs (up- and down-regulated genes combined for each cell population and generation) except that DEGs of the P6 somatic cell population of the F1 male fetal testes were significantly enriched with the Biological Pathways GO terms *DNA-templated transcription, positive regulation of RNA polymerase II-driven transcription, skeletal muscle differentiation, response to endoplasmic reticulum stress, fat cell differentiation,* and *apoptotic process* (Figure 4b). Agreeing with these Biological Pathway terms, these DEGs were enriched for Molecular Function GO terms Sequence-specific *DNA binding transcription factors*. These results suggest that the in utero exposure of F1 males to TBT affected testicular somatic cell expression of transcription factors that may be involved in mesenchymal lineage differentiation to muscles or adipocytes although such effects did not persist in the F3 testes.

**Figure 5:**
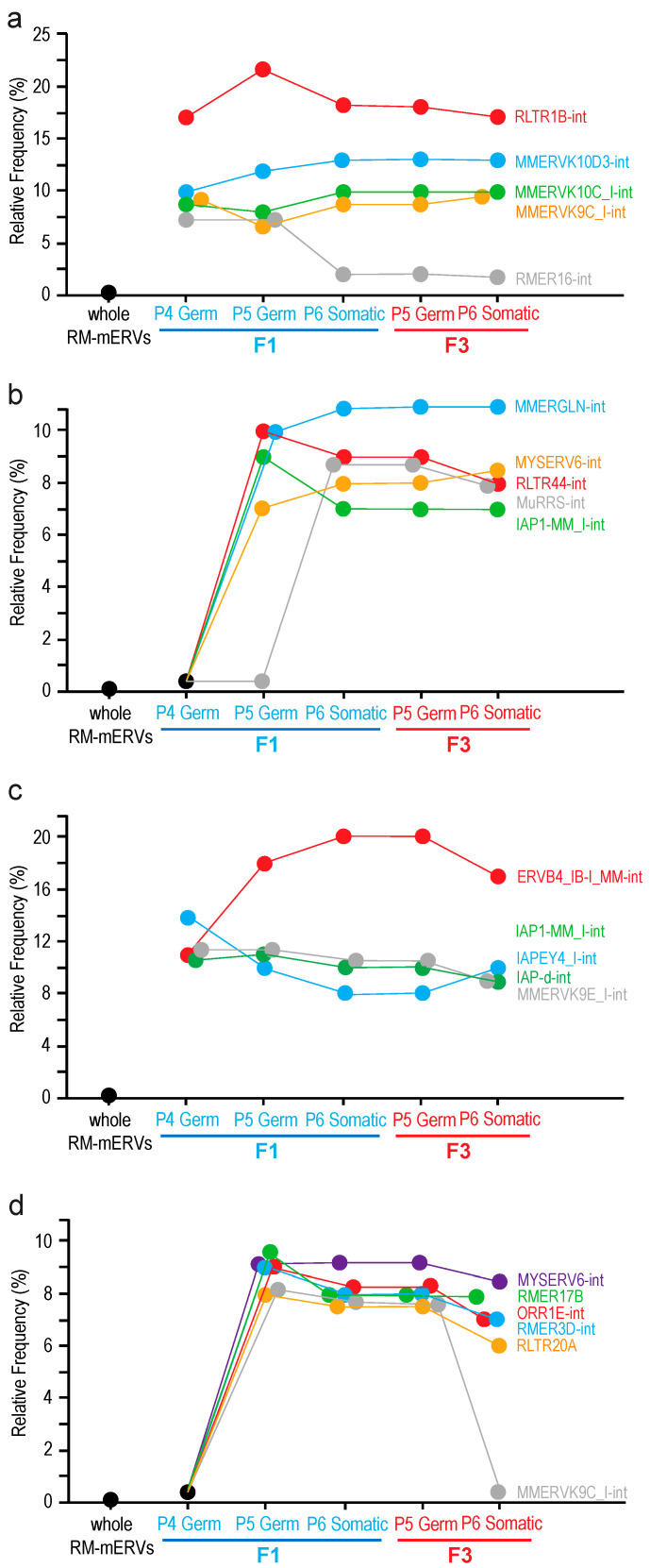
Differentially expressed mERVs. mERVs differentially expressed between E18.5 fetal testicular cells in F1 or F3 offspring of the TBT- and DMSO-exposed F0 mothers. Statistically significant, differentially expressed mERVs (FDR < 0.05) in the FACS-enriched germline or somatic cells were identified. Relative frequencies of mERV species enriched beyond the frequencies expected from random sampling from the whole RepeatMasker (RM) registered mERVs are plotted. (**a**,**b**) mERVs up-regulated in the TBT-group offspring; (**c**,**d**) mERVs down-regulated in the TBT-group offspring. Panels (**a**,**c**) show mERVs whose expression in P4 germline cells was affected by F0 exposure to TBT whereas mERVs shown in panels (**b**,**d**) are not affected.

**Effects of F0 exposure to TBT on expression of the endogenous retroviruses in fetal testicular cells of the offspring.** In animal cells, expression of RNA transcripts from endogenous retroviruses is suppressed transcriptionally by epigenetic mechanisms involving formation of heterochromatin as well as post-transcriptionally by mechanisms involving the piRNA. Expression of endogenous retrovirus RNA in germline cells is often affected by exposure to biologically active compounds such as endocrine disruptors as we previously reported [19]. Using our RNA-seq data and the ERVmap pipeline [20] with modifications for its application to examine expression of mERVs, we identified mERVs whose RNAs were differentially expressed between TBT and DMSO testes of F1 or F3 testicular cells (Figure 5). We first identified differentially expressed, individual mERVs registered in the RepeatMasker file [21] for the GRCm38/mm10 mouse reference genome sequence as the LTR family repeat sequences using a relatively strict cutoff criterion (FDR < 0.05). Then, frequencies of the 679 species of mERVs in the list of all individual, differentially expressed mERVs were counted. Frequencies of mERV species randomly extracted from the whole RepeatMasker-registered mERVs were calculated by a computational simulation. Finally, differentially expressed mERV species that were enriched beyond the frequencies expected from random extraction from the whole RepeatMasker-registered mERVs were chosen. Using this approach, we identified mERV species whose RNA expression was up-regulated by F0 exposure to TBT in both F1 and F3 testicular cells (Figure 5). We identified RLTR1B-int as the mERV by far most strongly enriched, up-regulated mERVs in both germline and somatic cells and in both F1 and F3 generations (Figure 5a). Other up-regulated mERVs were the Mus musculus ERVK species MMERVK9C_I-int, MMERVK10C_I-int, and MMERVK10D3-int (Figure 5a). Similar up-regulated mERV species but not up-regulated in P4 germline cells were MMERGLIN-int, MYSERV6-int, RLTR44-int, MuRRS-int, and IAP1-MM_I-int (Figure 5b). We also identified RMER16-int as a mERV up-regulated only in the F1 germline cells but not in the F1 testicular somatic cells or any F3 testicular cells (Figure 5a).

We also detected down-regulated mERVs (Figure 5c,d). The most enriched, down-regulated mERV was ERVB4_IB-I_MM-int (Figure 5c). Other down-regulated mERVs included the IAP species IAP1-MM_I-int, IAPEY4_I-int, and IAP-d-int, as well as an ERVK species MMERVK9E_I-int (Figure 5c). Down-regulated genes not repressed in P4 germline cells were RMR17B, RMR3D-int, ORR1E-int, and RLTR20A (Figure 5d). MMERVK9C-int, which was enriched for TBP up-regulated mERV species, was also enriched for down-regulated mERVs although its enrichment was limited to F1 and F3 P5 germline cells and F1 P6 somatic cells (Figure 5d).

**Figure 6:**
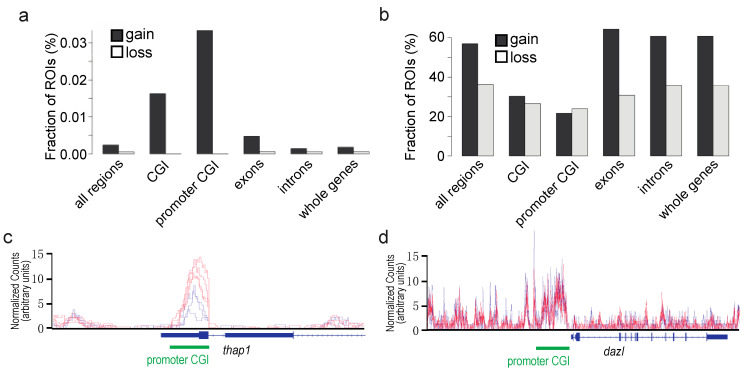
Effects of F0 exposure to TBT on DNA methylation in E18.5 F1 fetal testicular somatic cells determined by MBD-seq. (**a**,**b**) Changes in DNA methylation at representative genomic regions of interest (ROIs) in TBT-F1 somatic cells compared to DMSO-F1 somatic cells; (**a**) changes in differentially methylated regions, (**b**) changes in the whole genome; (**c**,**d**) representative DNA methylation profiles at a differentially methylated (**c**) or not, (**d**) promoter-associated CpG islands. Normalized methylation scores calculated for DMSO-F1 (blue) or TBT-F1 (red) testicular somatic cells isolated from three independent litters of fetuses are shown.

**Effects of F0 exposure to TBT on gDNA methylation in fetal testicular cells of the offspring.** We previously reported effects of the F0 dam exposure to TBT on gDNA methylation in white adipose tissues of the F4 male offspring [5]. Although in the current study we attempted to determine effects of the F0 exposure on gDNA methylation in E18.5 fetal testicular germline cells, the MBD-seq approach to detect differentially methylated regions (DMRs) has proven technically challenging primarily due to limited amounts of cells. Detection of DMRs in the P6 somatic cells of F3 male fetuses was also largely unsuccessful. PCA of MBD-seq data (Figure S1c,d) showed poor separation of TBT- and DMSO-groups in both F1 and F3 generations, indicating that F0 exposure to TBT did not strongly impact the global DNA methylation. However, we were able to identify statistically significant DMRs in the P6 testicular somatic cells of F1 male fetuses (FDR < 0.05). These DMRs were strongly enriched at promoter-associating CpG islands (CGIs) as CpG sites that gained cytosine methylation in the TBT-F1 P6 cells compared to DMSO-F1 P6 cells (Figure 6a; genes whose promoter-associating CpG islands covering the transcription start site are differentially methylated are shown in Table S2). In contrast, when statistically insignificant DMRs (FDR < 0.5) were examined similarly, the enrichment of promoter CGIs with a strong bias to gained methylation was lost (Figure 6b), supporting the specificity of TBT-induced gain in CpG methylation at promoter-associated CGIs. An example of a gene whose promoter-associated CGI was hyper-methylated in TBT-F1 P6 cells compared to DMSO-F1 P6 cells is shown as Figure 6c. An example of promoter-associated CGI whose expression in TBT-F1 P6 cells was not affected by F0 exposure to TBT is shown as Figure 6d.

## 3. Discussion

Evidence is accumulating that obesogenic effects of EDCs from gestational exposures can be transgenerationally transmitted to subsequent generations [3,22]. We previously showed that exposure of pregnant mouse dams to environmentally relevant doses of TBT initiated a transgenerationally inherited predisposition to diet-induced obesity in not only the directly exposed generations (i.e., F1 and F2) but also generations never exposed to the chemical (F3 and F4) [5,6,7]. In our model presented in the current study as well as the preceding report [5], pregnant F0 mice were exposed to a low dose of TBT (below the established no observed adverse effect level, NOAEL) via the drinking water. Thus, our model is uniquely relevant to realistic environmental exposure of humans to this environmental pollutant [23].

Reproducing our prior observations using the C57BL/6 mice [5], our current study showed that exposure of pregnant F0 dams of the OG2 transgenic mice [10] backcrossed to the C57BL/6 genetic background to a low dose of TBT caused diet-induced obesity in F3 generation males. When fed with an HF diet, TBT-F3 males accumulated more body fat than DMSO-F3 males. In contrast, when they were fed with a standard chow, body fat composition of TBT-F3 males was not significantly different from that of DMSO-F3 males (Figure 2b,c) [5]. As in our previous studies, HF diet-induced body fat accumulation was not significantly different between TBT-F3 and DMSO-F3 females (Figure 2b,c) [5]. Many other rodent models of obesity caused by in utero exposure to EDCs (such as F1 male obesity caused by perinatal exposure to Bisphenol A as we previously described [24]) showed increased total body weight as the primary phenotype. In our TBT model, total body weight was not affected by F0 exposure to TBT because body fluid and lean compositions were reduced upon HF diet challenge more greatly in TBT-F3 males than in DMSO-F3 males (Figure 2d–g). This increased body fat accumulation in HF diet-fed TBT-F3 males is reminiscent of human *normal-weight obesity (NWO)*, which is defined as having a normal body mass index but a high fat mass. Although individuals with NWO represent an underdiagnosed and understudied group of patients presenting metabolic disorders, there is growing interest in NWO as their high risk for cardiometabolic morbidity and mortality [12,25,26,27]. Our model may provide unique opportunities to examine mechanisms of diet-dependent development of NWO. The male-specific vulnerability to EDC-induced obesity observed in our current and previous studies [5,6,24] may reflect significantly distinct endocrine mechanisms regulating energy metabolism.

Our preceding studies identified protein-coding genes differentially expressed between TBT- and DMSO-exposed F3 and/or F4 male offspring in white adipose tissue, and GO analysis revealed their significant enrichment in biological function terms relevant to energy metabolism [5,6]. Our current study did not detect any statistically significant, global transcriptomic impact of F0 exposure to TBT on F1 or F3 E18.5 fetal testicular cells (Figure 2) or GO term enrichment for DEGs in the germline cells (Figure 3 and Figure 4). These negative outcomes are not surprising. Even when the F0 exposure to TBT could alter epigenetic mechanisms regulating expression of obesity-relevant genes in adult fat or liver as we previously demonstrated, such epigenetic alterations may not necessarily affect expression of these genes in fetal testes (which may not express these genes at all). Since we did not observe differences in fecundity or sex ratio in offspring of the TBT- and DMSO-exposed F0 mothers, F0 exposure to TBT did not cause drastic changes in testes. Our RNA-seq data confirmed the absence of sizable non-specific damages in viability or development of fetal testicular germline and somatic cells even in the directly exposed fetuses. DEGs in F1 fetal testicular somatic cells showed significant enrichment of GO terms for expression of DNA sequence-specific transcription factors (Figure 4b,c), suggesting a significant impact of the in utero exposure to TBT on development of testicular somatic cells, including Sertoli cells and Leydig cells. The absence of DEGs in fetal testicular somatic cells of F3 males (Figure 4a) supports the notion that TBT affects transcription in directly exposed testicular somatic cells but that such effects may not be transgenerationally sustained in the same types of cells not directly exposed to the chemical. Although we were able to detect an increase in DNA methylation in promoter-associated CGIs in TBT-exposed fetal testicular somatic cells in the F1 generation, such changes were largely undetectable in the F3 generation (Figure 6).

Our preceding studies provided evidence that perinatal exposure to TBT introduced potentially persistent epigenetic changes in the exposed male germline cells [5,7]. Such epigenetic changes were not gene-centric but rather implied relatively large-scale alterations in the global genome structure [5,7]. In this context, our current data demonstrating persistent changes in RNA expression from mERVs are significant (Figure 5). Expression of endogenous retroviruses in animal cells is suppressed primarily by epigenetic mechanisms involving DNA methylation and subsequent heterochromatin formation. For example, the microrchidia (MORC) family ATPase MORC1 is expressed specifically in fetal male germline cells and plays critical roles in gene silencing and chromatin compaction widely in eukaryotes [28]. Mammalian MORC1 is critical for suppression of transposons (including mERVs) in mouse fetal germline cells [15]. It is intriguing that four out of nine mERVs (RLTR1B, MMERVK10C, MURRS-int, and MMERGLN) whose expression was up-regulated transgenerationally in both F1 and F3 germline and somatic cells by F0 dam exposure to TBT in the current study were previously identified as representative mERVs whose DNA is very strongly demethylated in mouse fetal germline cells lacking MORC1 [15]. The persistent relaxation in the epigenetic suppression of these mERVs in TBT-exposed testicular cells of the F1 and F3 generations supports the notion that TBT exposure may have disrupted the MORC1-dependent epigenetic suppression of mERVs. Such apparently persistent relaxation in the epigenetic suppression of transposons might underlie the transgenerational inheritance of the obesity predisposition. Alternatively, up-regulation of mERVs may not be the direct cause of the transgenerational phenotype but rather a marker reflecting genome-wide alterations in heterochromatin structures or distributions that are not strongly affecting expression of many protein-coding genes in the fetal testicular cells. Expression of some mERVs—including three IAP species IAP_MM_I-int, IAPEY3_I-int, and IAP-d-int—was persistently suppressed, implying possible heterogeneity in the impact of TBT on the genome structure. Further studies will be necessary to elucidate details of the basis of these altered and persistent expression of mERVs after perinatal exposure to TBT.

In summary, our current study reproduced our previous observation that exposure of pregnant F0 dams of the OG2 transgenic mice to TBT caused transgenerationally transmittable predisposition to HF diet-inducible, normal-weight obesity specifically in male offspring. Transcriptomic and DNA methylome analyses did not detect transgenerationally persistent changes in mRNA expression of protein-coding genes or CpG methylation at promoter-associated CGIs in fetal testes. However, we were able to identify transgenerationally persistent changes in RNA expression from several representative mERVs, including species known to be repressed in mouse male germline cells by an epigenetic mechanism involving the chromatin compaction protein MORC1. These observations support a model in which F0 exposure to TBT may leave global epigenetic alterations that can be transmitted over generations in germline cells.

## 4. Materials and Methods

**Animal maintenance and exposure.** The OG2 transgenic mice [B6;CBA-Tg(Pou5f1-EGFP)2Mnn/J], which express EGFP specifically in the germline cells driven by the *Pou5F1* promoter and distal enhancer [10], were purchased from The Jackson Laboratory (strain number 004654) and maintained in the C57BL/6J background. All mice were housed in a temperature-controlled (21–22 °C) barrier room at the Massachusetts General Hospital Center for Comparative Medicine with a 12 h light, 12 h dark cycle. Water and food were provided ad libitum, and animals were treated humanely and with regard for alleviation of suffering. All procedures conducted in this study were approved by the Massachusetts General Hospital Institutional Animal Care and Use Committee. All tissue harvesting was performed with the dissector blinded to which groups the animals belonged.

Exposure and animal breeding were performed as we previously described [5]. Female OG2 mice (25 females per treatment group) were exposed to 50 nM TBT or 0.1% DMSO via drinking water containing 0.5% carboxymethyl cellulose. Exposure started from 7 days prior to mating and continued throughout lactation until 3 weeks after delivery (See Figure 1). Sires were not exposed to TBT or DMSO. Litter size and sex ratio were not affected by exposure to TBT. F1 males and females belonging to the same exposure group (i.e., TBT or DMSO) were mated to obtain F2 animals. Siblings were not mated, and a male was mated with not more than one female belonging to each litter. F2 mice were bred similarly to obtain F3 animals. A subset of pregnant F0 and F2 dams were randomly chosen and subjected to collection of E18.5 F1 and F3 male fetuses.

**Dietary challenge.** F3 mice were fed with a standard chow (Prolab Isopro RMH 3000 5P75; 14.0% calories were provided by fat) until 19 weeks of age. Randomly selected mice were then challenged with a high-fat (HF) diet (Picolab mouse diet 20 5058; 21.6% calories were provided by fat) from weeks 19 until 25. Total body weight was measured before and after the HF diet challenge. Because metabolizable energy amounts of the standard and the HF chows were 3.20 and 3.45 kcal/gram, respectively, the HF chow was not considered as high-energy diet.

**Body composition.** Body composition of F3 mice was determined before and after HDF challenge using a Bruker Minispec nuclear magnetic resonance analyzer (Bruker, Billerica, MA, USA). Animals were placed in a clear, plastic cylinder (50 mm diameter) and kept immobile by gentle insertion of a plunger. The tube was then lowered into the nuclear magnetic resonance instrument for the duration of the scan (less than 2 min).

**Isolation of fetal testicular cells and nucleic acids.** Testes were isolated from E18.5 male fetuses of the F1 and F3 generations by microscopic dissection and digested with 1x trypsin/EDTA to prepare single cell suspensions. Undigested debris was removed from the cell suspensions by passing through 40 μm cell strainers, and cells were subjected to FACS separation of the EGFP-positive germline cells and EGFP-negative somatic cells with GFP and side scatter gates as previously described [10]. Total RNA and genomic DNA (gDNA) were isolated from the FACS-separated germline and somatic cells using AllPrep Micro kit (Qiagen). Integration of total RNA was evaluated using Agilent Tapestation using RNA high sensitivity tapes and confirmed intact (RIN > 8.0).

**RNA-seq transcriptomic analysis.** RNA-seq was performed as we previously described [29]. Briefly, cDNA was synthesized from total RNA using the SMARTer Ultra Low Input RNA Kit for Sequencing (TAKARA Bio, San Jose, CA, USA) with an oligo(dT) primer provided in the kit and subjected to construction of Illumina deep sequencing libraries using the Low Input DNA Library Prep Kit. Libraries were sequenced using an Illumina NextSeq500 deep sequencer with High Output Kit v2 (Illumina) to obtain 75 nt + 75 nt paired-end FASTQ reads, which were subjected to quality control analysis using the fastQC tool (Babraham Institute, Cambridge, UK). After adaptor sequences and low-quality reads (<30) were trimmed off using the Trim Galore! tool, FASTQ reads were aligned to the GRCm38/mm10 mouse reference genome using the STAR aligner software [30] to obtain BAM files.

To evaluate expression of protein-coding mRNA transcripts, aligned reads in the BAM format were assigned to exons of the mm10 gene model and counted using the Bioconductor package Rsubread [31]. The mRNA expression counts were normalized using the negative binominal trimmed mean of M-values (TMM) method implemented by the Bioconductor package edgeR [32]. The normalized counts were subjected to unsupervised hierarchical clustering analysis and visualization using Cluster [33] and Java TreeView [34]. Differentially expressed genes (DEGs) were identified using the generalized linear model likelihood ratio test implemented by edgeR [32] with a criterion of false discovery rate (FDR) < 0.1 and subjected to gene ontology (GO) analysis using the DAVID server [35].

RNA expression from mouse endogenous retroviruses (mERVs) was evaluated using the ERVmap pipeline [20] with modifications as we previously described for quantitative detection of RNA transcripts of chicken endogenous retroviruses [19]. The original ERVmap was developed for quantitative detection of RNA transcripts expressed from a selected subset of human endogenous retroviruses [20]. To evaluate expression of mERVs, we replaced its list of human endogenous retroviruses with mERVs extracted as LTR repetitive sequences from the RepeatMasker file [21,36] of the GRCm38/mm10 mouse reference genome. The mERV counts were normalized using the TMM algorithm [37]. Differentially expressed mERVs were identified using the generalized linear model likelihood ratio test implemented by edgeR [32] with a criterion of FDR < 0.05. mERV species of the differentially expressed mERVs were counted separately for mERVs up- and down-regulated in the F1 or F3 animals that are descendants of the TBT-exposed F0 mothers compared to the descendants of the DMSO-exposure F0 mothers.

**MBD-seq DNA methylome analysis.** MBD-seq was performed as we previously described [5]. Briefly, sonicated gDNA fragments (100–200 bp, 500 ng) were subjected to methylated DNA enrichment using the MethylMiner kit (Life Technologies, Waltham, MA, USA) followed by deep sequencing library construction using the NEBNext Ultra kit (New England Biolabs). A library constructed from similarly sonicated mouse gDNA without methylation enrichment was used as input control. Statistical evaluation of TBT-dependent alterations in gDNA methylome was performed using R/Bioconductor package QSEA [38]. Numbers of the independently generated samples for F1-DMSO, F-TBT, F3-DMSO, and F3-TBT were 3, 4, 5, 7, respectively.

**Statistics.** Statistical significance of difference in averages between the DMSO group and the TBT group was tested independently for each experimental condition (male versus female; standard versus high-fat diet) using two-tailed Student’s *t*-test. Because *F*-tests did not support equal variance of the four groups in each sex (which is a required assumption of ANOVA or Tukey’s test), the *t*-statistic was corrected using the Welch’s formula for heteroscedastic data pairs. Differentially expressed protein-coding mRNAs or mERVs were identified by the generalized linear model likelihood ratio test with multiple comparison correction for calculation of FDR values [32,37]. Statistical evaluation of DMRs identified from the MBD-seq data was performed using the R/Bioconductor package QSEA [38]. Principal Component Analysis of deep sequencing data was performed using the R function prcomp and visualized using the R package rgl.

## Data Availability

The deep sequencing data presented in this study are available at Gene Expression Omnibus, reference number GSE191199.

## Supplementary Materials

The following are available online at https://www.mdpi.com/article/10.3390/metabo12020095/s1, Figure S1: Principal Component Analysis (PCA) of RNA-seq and MBD-seq data, Table S1: Differentially Expressed Genes, Table S2: Genes Associated with Differentially Methylated Regions.
