# Supplementary material for: Transgenerational Transcriptomic and DNA Methylome Profiling of Mouse Fetal Testicular Germline and Somatic Cells after Exposure of Pregnant Mothers to Tributyltin, a Potent Obesogen
Source: Metabolites. 2022 Jan 20;12(2):95. doi: 10.3390/metabo12020095 (PMC8874857; doi:10.3390/metabo12020095)

Figure S1. Principal Component Analyses (PCA) of RNA-seq and MBD-seq data.

PCA of RNA-seq (**a, b**) and MBD-seq (**c, d**) are presented as 3-D (**a, b**) or 2-D (**c, d**) plots. For RNA-seq, all data from F1 and F3 generations are combined and presented for (**a**) generations and testicular cell types or (**b**) exposure. MBD-seq data of the testicular somatic cells are presented for (**c**) F1 and (**d**) F3 generations.

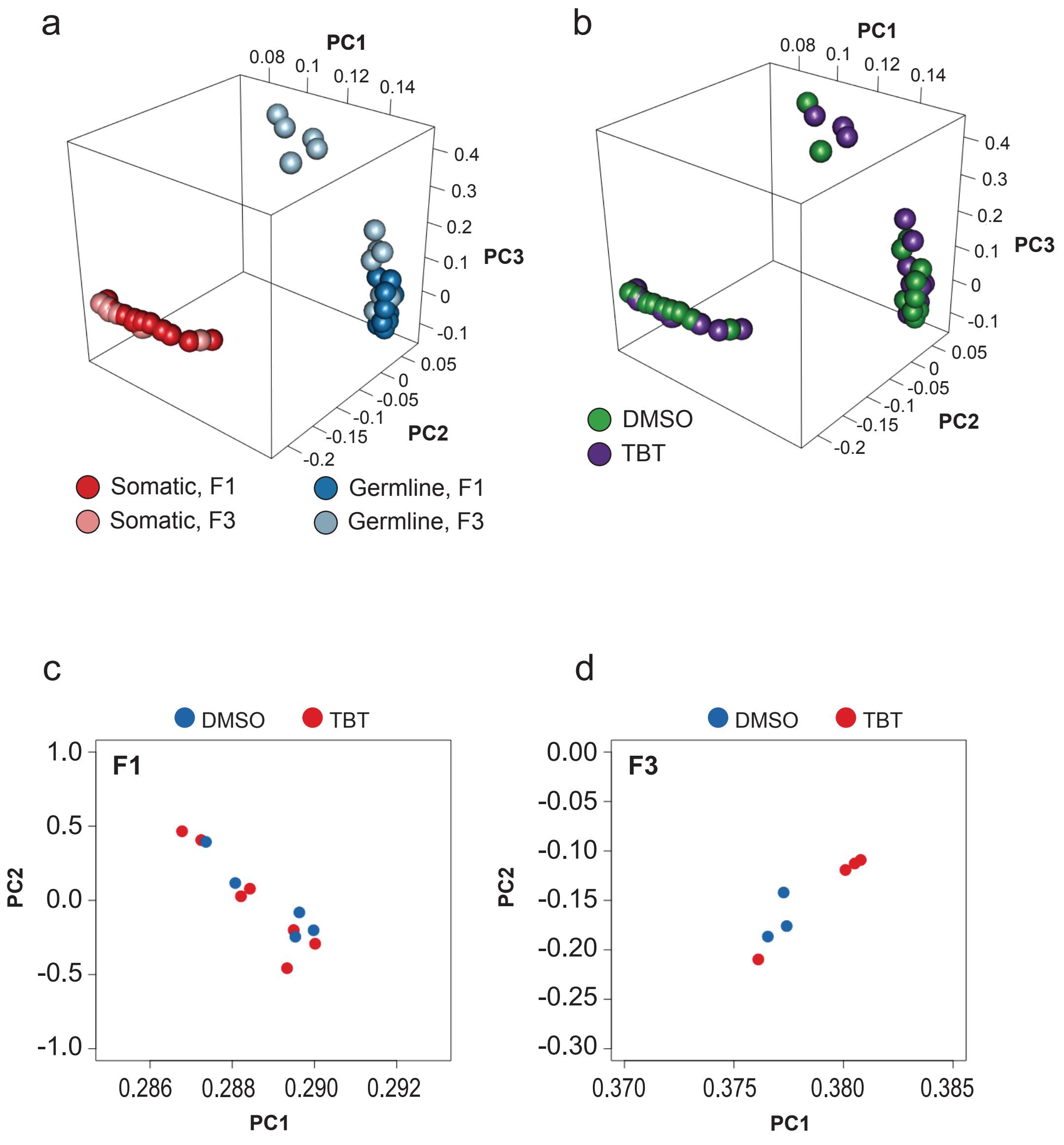

Supplement: Supplementary file 1 [file metabolites-12-00095-s001.zip › Figure S1 Principal Component Analysis of RNA-seq and MBD-seq data.pdf]
